# Impact of central and peripheral TRPV1 and ROS levels on proinflammatory mediators and nociceptive behavior

**DOI:** 10.1186/1744-8069-6-46

**Published:** 2010-08-06

**Authors:** Karin N Westlund, Mikhail Y Kochukov, Ying Lu, Terry A McNearney

**Affiliations:** 1 Department of Physiology, University of Kentucky, Lexington, KY 40536-0298, USA; 2 Department of Neuroscience & Cell Biology University of Texas Medical Branch, Galveston, TX, 77555-1069, USA; 3 Department of Internal Medicine, University of Texas Medical Branch, Galveston, TX 77555-1069, USA; 4 Department of Microbiology & Immunology, University of Texas Medical Branch, Galveston, TX 77555-1069, USA

## Abstract

**Background:**

Transient receptor potential vanilloid 1 (TRPV1) channels are important membrane sensors on peripheral nerve endings and on supportive non-neuronal synoviocytes in the knee joint. TRPV 1 ion channels respond with activation of calcium and sodium fluxes to pH, thermal, chemical, osmotic, mechanical and other stimuli abundant in inflamed joints. In the present study, the kaolin/carrageenan (k/c) induced knee joint arthritis model in rats, as well as primary and clonal human synoviocyte cultures were used to understand the reciprocal interactions between reactive nitroxidative species (ROS) and functional TRPV1 channels. ROS generation was monitored with ROS sensitive dyes using live cell imaging *in vitro *and in spinal tissue histology, as well as with measurement of ROS metabolites in culture media using HPLC.

**Results:**

Functional responses in the experimental arthritis model, including increased nociceptive responses (thermal and mechanical hyperalgesia and allodynia), knee joint temperature reflecting local blood flow, and spinal cord ROS elevations were reduced by the ROS scavenger PBN after intraperitoneal pretreatment. Increases in TRPV1 and ROS, generated by synoviocytes *in vitro*, were reciprocally blocked by TRPV1 antagonists and the ROS scavenger. Further evidence is presented that synoviocyte responses to ROS and TRPV1 activation include increases in TNFα and COX-2, both measured as an indicator of the inflammation *in vitro*.

**Conclusions:**

The results demonstrate that contributions of ROS to pronociceptive responses and neurogenic inflammation are mediated both centrally and peripherally. Responses are mediated by TRPV1 locally in the knee joint by synoviocytes, as well as by ROS-induced sensitization in the spinal cord. These findings and those of others reported in the literature indicate reciprocal interactions between TRPV1 and ROS play critical roles in the pathological and nociceptive responses active during arthritic inflammation.

## Background

An emerging awareness in the field of neuroimmunity is the close physiological tie between the activation of transient receptor potential vanilloid type 1 (TRPV1) ion channels and ROS formation [1-6]. Reactive nitroxidative species (ROS - superoxide anion, hydrogen peroxide, hydroxyl, nitric oxide and peroxynitrite radicals) are highly reactive molecules which in low concentrations (5-10 μM) are required for many cellular regulatory mechanisms. The TRPV cation channels are well known activators of nociceptive responses and have also been implicated in direct mediation of inflammatory events [7]. Previous studies have shown reduced sensitization responses in the complete Freund's adjuvant (CFA) hindpaw inflammation model with TRPV1 antagonists in physiological dose ranges [8,9] as well as in TRPV1 knockout mice [10]. The TRPV1 agonist, capsaicin, is in clinical use in concentrations sufficient to inactivate C fiber nerve terminal endings producing analgesic effects.

Tissue injury rapidly initiates oxidative stress leading to intracellular production of excessive ROS levels, such as the reactive molecule H_2_O_2 _[11], which increases the likelihood of extracellular spread. Evidence supports the potential for ROS themselves to activate TRPV1 cation channels after conformational change of this integral membrane protein [12], directly increasing plasma membrane permeability to calcium ions [12,13]. For example, footpad injection of H_2_O_2 _has been shown to produce pain related behaviour through TRPV1 dependent and independent mechanisms [14]. ROS activation of TRPV1 on vascular membranes also increases local blood flow [15]. Thus, increasing and persisting levels of ROS have a dramatic impact on tissue and cellular function associated with inflammation.

The TRP superfamily of ion channels is implicated in the pathogenesis of arthritis. Their multimodal activation occurs with decreasing pH, osmolar changes, mechanical pressure, heat, cold, and noxious biochemicals, stimuli all very relevant to joint physiology and pathology. While it is well known that TRPV1 channels are found on primary afferent nerves in the periphery [16,17], we have recently characterized and described several functional TRP channels (TRPV1, TRPV4 and TRPA1) present on human synoviocytes [18,19]. Synoviocytes line the joint capsules and in healthy individuals produce the cushioning synovial fluid. As immunoresponsive cells they also secrete inflammatory mediators in inflamed and injured joints.

The current study was initiated to gain a better appreciation of reciprocal interactions between TRPV1 channels and ROS generation that contribute to neurogenic/neuroimmune inflammation and nociceptive responses.

## Results

To examine ROS and TRPV1 interactions, two arthritis models were used in the present study including (a) *in vivo *inflammation of the knee joint in rats induced by injecting 3% kaolin and 3% carrageenan (k/c) directly into the knee joint injection and (b) *in vitro *human clonal type B synoviocytes (SW982) and primary synoviocytes harvested from clinic patients with active arthropathies [19]. The first approach was to demonstrate whether modulation by ROS scavengers would reduce the hypersensitized responses in animals with k/c inflamed knee joints. We examined not only the role of ROS in inflammation through the generation of sensitized behavioral responses, but also the ability of ROS scavengers to reduce the generation of ROS in the spinal cord dorsal horn.

### ROS Scavenger Reduces Knee Joint Temperature But Not Circumference

Baseline measurements of knee joint temperature, circumference, and nociceptive behaviors were conducted in male Sprague Dawley rats (n = 8). Each animal served as its own control in a repeated measures analysis of variance experimental design (Mann Whitney U post-hoc testing) with the significance level set at p < 0.05. The rats received intrathecal administration of the ROS sensitive dye, dihydroethidium (DHE, 50 μl of 100 μM). The following day, half of the animals received an intraperitoneal (i.p.) injection of the ROS scavenger PBN (100 mg/kg) and half received saline i.p. The left knee joints (condyle fossa) of the hindlimbs of the anesthetized rats were injected 1 h later with 3% carrageenan (plant product and irritant) and 3% kaolin (clay silt) (100 μl in sterile saline) to produce an acute inflammation confined to the one knee joint.

**Figure 1:**
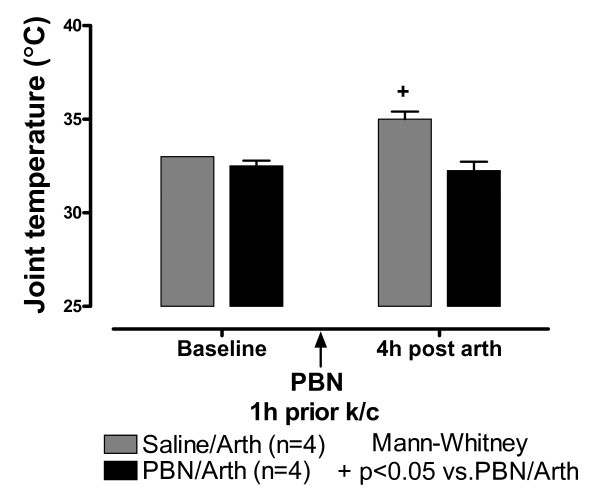
**Knee Joint Temperature Increase is Blocked by ROS Scavenger PBN**. Right and left knee joint temperature (°C) at baseline and 4 h after k/c induction of left knee joint inflammation (Arth) in rats pre-treated i.p. with saline (n = 4) or the ROS scavenger, PBN (n = 4). The PBN provided protection from significant increase in knee joint temperature typical of this arthritis model. Temperature increase in both knees is indicative of a centrally mediated "mirror" effect on regional blood flow. Core body temperature measured with a rectal thermometer was not increased over baseline.

Four hours after the k/c injection, the inflamed knee joint produced an ipsilateral abnormal stance with curled toes and foot eversion at rest. There was a slightly noticeable limp when animals moved slowly. There was no discernable difference from controls in rapid locomotion. Knee joint circumference increased 1 cm on average (4-8 h) on the injected left knee but not on the uninjected knee as previously reported [20]. Skin temperature was elevated over baseline in both knees indicating mirror blood flow increase despite negligible change in joint circumference in the uninjected knee joint (Fig. 1, hatched bars, p < 0.05; 4 h).

In animals pre-treated i.p. with ROS scavenger, PBN, circumference of the injected knee in animals was also increased by a similar amount (0.78 cm ± 0.1 s.e.m., 4-8 h). However, skin temperature of both knees remained at baseline 4 h after induction of inflammation (Fig. 1, black bars). Core body temperature was not increased over baseline in any of the animals (data not shown).

### ROS Scavenger Reduces Nocifensive Behaviors

#### Mechanical Allodynia and Hyperalgesia

Behavioral testing of mechanical sensitivity four hours after the induction of the knee joint inflammation was done by applying von Frey filaments of two strengths (10 mN and 47 mN) upward through a wire mesh tabletop onto the footpad of the animals. Ten bending strokes (5 sec) were applied to different sites on the footpad of the hindlimbs. The pressure of the 10 mN filament was barely detectable to human skin. Behavioral testing on the bottom of the footpad clearly differentiated the sensitized responses as secondary mechanical allodynia in response to the weaker 10 mN stimuli and secondary mechanical hyperalgesia for the stronger 47 mN stimuli.

**Figure 2:**
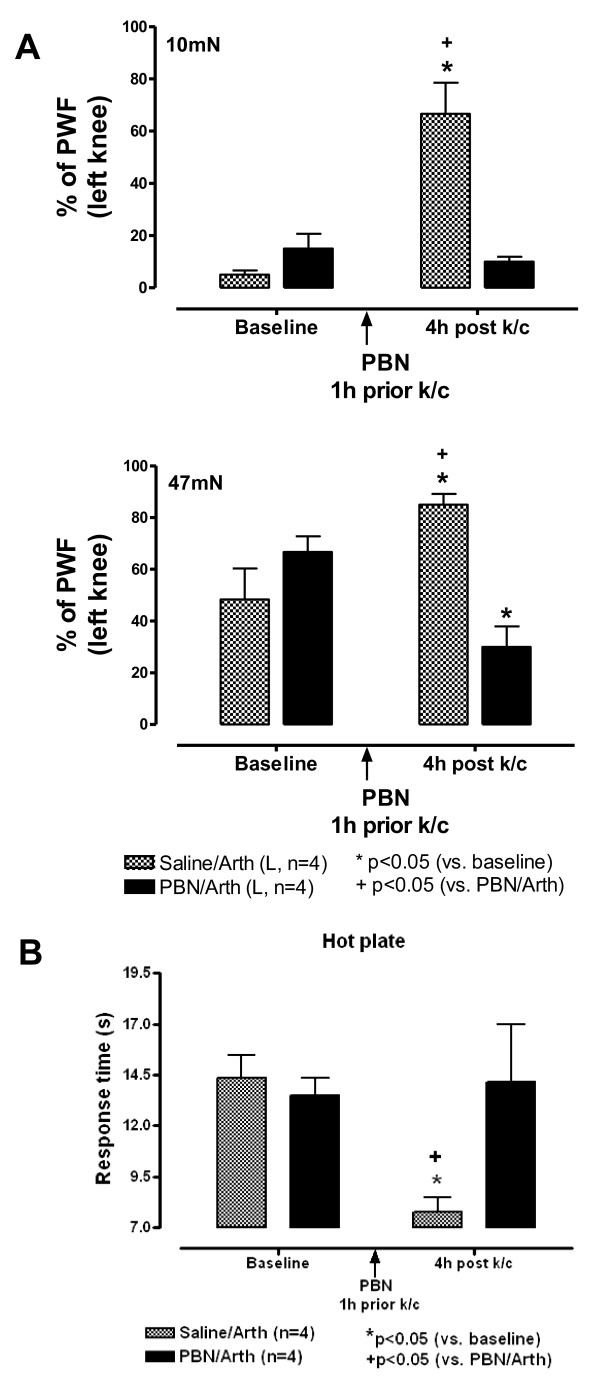
**Pain Related Behaviors are Blocked by PBN**. **A - Mechanical Hyperalgesia/Allodynia are Blocked by ROS Scavenger PBN**. Responses to mechanical stimulation with two strengths of von Frey filaments are shown for rats at baseline and 4 h after k/c injection into the left (L) knee joint. Secondary mechanical allodynia is tested on the footpad with the weak 10 mN stimulus (upper bar chart), and secondary mechanical hyperalgesia is observed with the stronger 47 mN stimulus (lower bar chart). Percent paw withdrawal frequency (PWF) is significantly increased at 4 h after i.p. pre-treatment with saline (n = 4). The hypersensitive responses are blocked by the ROS scavenger, PBN, injected intraperitoneally 2 h before induction of the knee joint inflammation (n = 4). **B - Hotplate Response is Blocked by ROS Scavenger PBN**. Response times in seconds (s) are shown for the hotplate test at baseline and five hours after k/c induced left (L) knee joint arthritis (Arth) in rats pre-treated with saline (n = 4) or ROS scavenger, PBN (n = 4). The PBN pre-treatment administered i.p. 1 h prior to induction of arthritis prevented development of secondary thermal hyperalgesia observed in the saline treated group.

The percent of responses to strokes applied to the footpad on the side with the inflamed knee were significantly increased in animals pre-treated with saline compared to their own baseline responses. The percent responses increased from 5 ± 1.7% at baseline to 66.6 ± 11.9% (p < 0.01) at 4 h post (Fig. 2A). Responses were unchanged on the footpad of the uninjected side.

Pre-treatment with ROS scavenger PBN eliminated the sensitized responses. The level of sensitivity was not significantly altered compared to baseline in animals with inflamed knees pretreated with PBN (i.p.). The mean difference in percent response between the saline and PBN treatment groups at 4 h was significant (66.6 ± 11.9 vs 10 ± 1.9%, p < 0.01). For the stronger 47 mN bending force monofilament, the responses on the footpads in animals with inflamed knee joints were increased from 48.3+12% to 85+4.2%, p < 0.05). Pretreatment with PBN significantly reduced the response to mechanical stimulation below baseline (66.7 ± 6.1 to 30 ± 7.9%, p < 0.05). The response differences for saline versus PBN treated animals was also significant (85 ± 4.2 vs 30 ± 7.9, p < 0.01).

#### Heat Hyperalgesia

Sensitivity to heat was tested 5 h after induction of knee joint inflammation with the hotplate (50°C). Responses to the heat stimulus indicating a termination endpoint for the trial included paw flicking, licking or jumping at which point the rats were immediately removed from the hotplate. It was never necessary to employ the 20 sec maximal cut off time in this study. In animals pre-treated with PBN, response latency to thermal stimuli remained near baseline. Response times for the appearance of complex behaviours to the novel heated surface stimulus were recorded. Saline treated animals had significantly reduced response times compared to baseline for (14.4 ± 1.1 vs 7.8 ± 0.7 sec, p < 0.01) (Fig. 2B), while the response times of PBN treated animals were not different from their baseline (13.5 ± 0.8 vs 14.2 ± 2.8 sec, p>0.05). The response time was also significantly reduced at 4 h for saline treated animals versus the PBN treated animals with knee joint inflammation (7.8 ± 0.7 vs 14.15 ± 2.8 sec, p < 0.05). These sensitized responses to a novel heat stimulus indicate central sensitization mechanisms involving neurons in the dorsal horn of the spinal cord and higher pain processing centers.

### ROS Scavenger Reduces ROS Production in Spinal Cord of Rats with Inflamed Joints

**Figure 3:**
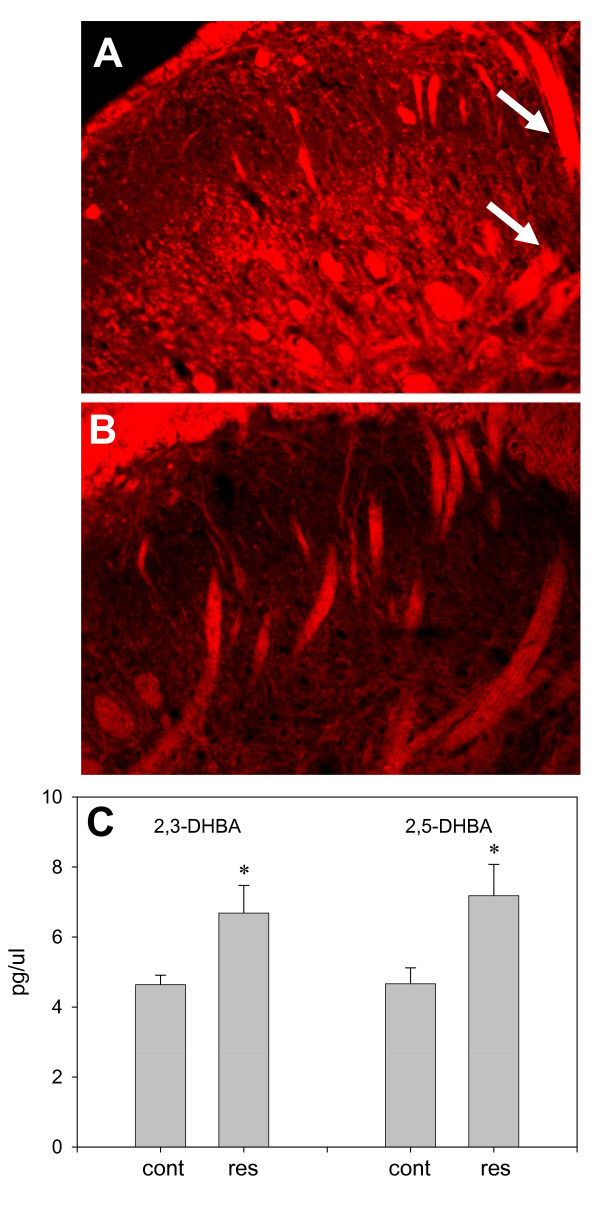
**ROS Generation**. **A - Lumbar Spinal Cord after K/C Induced Knee Joint Inflammation**. Cumulative generation of ROS is detected by conversion of the fluorescent indicator DHE in the dorsal horn of the L4 spinal cord segment in rats with k/c induced knee joint arthritis (6 h) (n = 4). **B - ROS Generation Is Blocked by ROS Scavenger PBN**. Much less DHE is observed in spinal cords of rats pretreated i.p. with ROS scavenger, PBN, 1 h prior to induction of knee joint inflammation with k/c (n = 4). Tissues were collected from paraformaldehyde perfused rats 6 h after induction of k/c knee joint arthritis. **C - TRPV1 Agonist Induces ROS in Human SW982 Synoviocytes**. Enhanced hydroxyl radical (HO•) production is induced by treatment of cultured clonal human SW982 synoviocytes with TRPV1 agonist resiniferatoxin. SW982 cells were incubated in bath saline (37°C) containing 2.5 mM salicylic acid and 100 nM resiniferatoxin (res) (n = 4) or vehicle (cont) (n = 4). After a 6 h incubation samples of extracellular solution were analyzed for stable ROS metabolites, 2,3- and 2,5-dihydroxybenzoic acid (2,3-DHBA and 2,5-DHBA), by HPLC with electrochemical detection as pictogram per microliter (pg/μl).*- indicates statistically significant difference (p < 0.05) between control and resiniferatoxin treated cells.

After induction of knee joint inflammation, ROS was observed in the spinal cord of animals pre-injected with the ROS indicator dye DHE (Fig. 3A). No fluorescent DHE was observed in the spinal cord of control animals. The ROS indicator was localized particularly in association with large cells and in the myelin sheaths of incoming afferent nerve fiber bundles (arrows). Localization in nerve bundles implies that ROS derived from oligodendroglia are important in glial/neuronal interactions during emerging inflammation induced pronociceptive states. Systemic pre-treatment with the PBN greatly reduced visualization of the ROS indicator in the spinal cord, concomitant with the decreased scores for nociceptive behaviour and joint temperature in animals with inflamed knee joints (Fig. 3B).

### TRPV1 Mediated ROS Generation: HPLC Measurement of ROS Metabolites from SW982 Synoviocytes *In Vitro*

TRPV1 channels have previously been characterized on clonal human type B SW982 synoviocytes using RT-PCR, Western blot and calcium mobilization after activation with TRPV1 agonists, capsaicin and resiniferatoxin [18,19]. In the present study, the clonal type B synoviocytes were utilized as an *in vitro *model of knee joint inflammation since these synoviocytes produce inflammatory mediators when activated with another channel activator, glutamate [21]. Unstable hydroxyl radicals (HO•) were induced by TRPV1 agonist resiniferatoxin in cultured clonal SW982 synoviocytes. While the unstable ROS were not measureable, metabolites 2,3- and 2,5-dihydroxybenzoic acid (2,3- and 2,5-DHBA) were measureable in the cell culture media by HPLC with electrochemical detection (Fig. 3C). Cell plates were incubated (37°C × 6 h, n = 4) in saline bath containing 2.5 mM salicylic acid with 100 nM resiniferatoxin or vehicle (n = 4). Samples of extracellular solution were taken and analyzed for ROS metabolites. The TRPV1 activation of the SW982 cells produced a statistically significant 1.68-fold increase in extracellular ROS metabolite 2, 5-DHBA (7.8 ± 0.9 pg/ml, p < 0.05) compared to control cells treated with the vehicle (4.6 ± 0.4 pg/ml).

### TRPV1 Mediated ROS Generation is Blocked by TRPV1 Antagonist and ROS Scavenger: Live Cell Imaging of ROS Sensitive Intracellular Dye

#### Clonal Human Type B SW982 Synoviocytes

**Figure 4:**
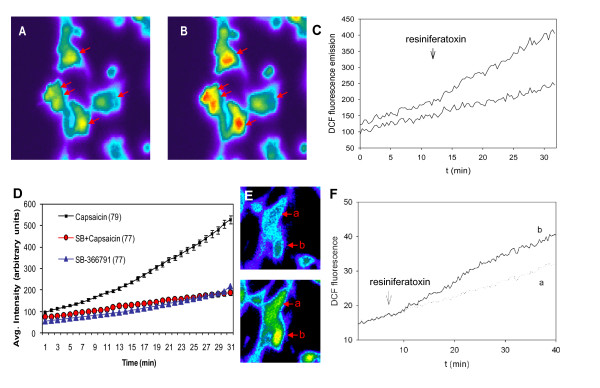
**TRPV1 Activates ROS Production - Live Cell Imaging**. **A-D Clonal SW982 Synoviocytes**. **A, B**- Live cell ROS imaging in SW982 synoviocytes before (**A**) and 15 min after (**B**) activation of TRPV1 with resiniferatoxin (100 nM). At least five of the cells in this view (arrows) demonstrated a significant increase in DCF fluorescence over time, reflecting a TRPV1-mediated oxidative burst. **C - **Quantification of fluorescent emission for the ROS response to resiniferatoxin over time (min) is shown in the plot. Cells were pre-loaded with 4 μM 5-(and-6)-chloromethyl-2', 7'-dichlorodihydrofluorescein diacetate (CM-H2DCFDA) for 20 min at room temperature. The cellular uptake and ROS converted 5-(and-6)-carboxy-2',7'-dichlorofluorescein (DCF) fluorescence was measured by live cell imaging for 30 min. Resiniferatoxin (100 nM) was added by bath perfusion for 4 min as indicated by the arrow. Graph shows typical fluorescence signal acquired from one responsive and one non-responsive cell out of 48 simultaneously recorded cells. **D - **Averaged DCF fluorescent emission responses to saline, capsaicin (1 μM), or capsaicin plus TRPV1 antagonist SB-366791 (30 μM) are shown in the plot (arbitrary units) over time (min). Fluorescent emission was significantly increased in cells activated with capsaicin. ROS induced fluorescent DCF emission in cells pre-treated for 10 min with the TRPV1 antagonist and then activated with capsaicin were not significantly increased over saline treated cells. Numbers of cells are indicated in parenthesis averaged from three experiments. **E-F TRPV1 Mediated ROS Production in Primary Human Synoviocytes from Two Patients**. Live cell imaging was used to visualize ROS generation over time (min) after stimulation with TRPV1 agonist resiniferatoxin and conversion of a ROS sensitive dye to fluorescent DCF in the synoviocytes. Primary human synoviocytes were acclimated 3 weeks in culture after harvesting from knee joints of patients with active arthropathies (osteoarthritis, pseudogout). Resiniferatoxin (100 nM) was added in bath perfusion for 4 min at the time indicated by arrow. Fluorescent image (**E**) and quantitative graph (**F**) show typical fluorescence signal acquired from one responsive (b) and one non-responsive (a) out of 85 simultaneously recorded cells. A total of 68 cells responded to the TRPV1 agonist out of 162 total cells imaged from the two patients.

Cellular ROS responses were visualized with live cell imaging in cells pre-loaded with 5- (and -6)-chloromethyl-2',7'-dichlorodihydrofluorescein diacetate, acetyl ester (CM-H2-DCFDA). The intracellular reactive oxygen species increases were detected by the oxidation of CM-H2-DCFDA and stimulated conversion to the fluorescent indicator 2'-7'-dichlorofluorocein (DCF). As illustrated in Figure 4, fluorescent cell intensity at baseline (Fig. 4A) was increased after addition of resiniferatoxin to the clonal human type B SW982 synoviocytes (Fig. 4B). The quantitative measure of the average fluorescent DCF emission in response to resiniferatoxin (100 nM, arrow) is shown plotted over time (Fig. 4C , upper trace). A 2-fold increase in ROS production generated by a representative synovial cell cultures after resiniferatoxin stimulation is shown for comparison to the response plotted for fluorescent DCF emission of a non-stimulated cell culture (Fig. 4C , lower trace) that displays only spontaneous oxidation of the DCF. The responses to resiniferatoxin were dose-dependent (not shown) and 56% of the cells were activated. The responses to resiniferatoxin were blocked by pretreatment with TRPV1 antagonist, capsaizepine (10 μM), and resulted in only 2% of the cells responding.

The responses to resiniferatoxin were also blocked by PBN (2 mM pretreatment for 2 h). Treatment with capsaicin also increased ROS in the SW982 cells (Fig. 4D, upper trace). Capsaicin responses were blocked by TRPV1 antagonists, capsaizepine and SB-366791 (30 μM). The averaged responses for all cells tested are shown (Fig. 4D, middle trace). Treatment with TRPV1 agonists capsaicin or resiniferatoxin yielded no responsive cells after pre-treatment with the ROS scavenger PBN (2 mM pretreatment for 2 h).

#### Human Primary Synoviocyte Cultures

The validity of the idea that TRPV1 activation generates ROS was also tested using human primary synoviocyte cultures harvested from clinical synovial fluid samples drawn from the knee joints of patients with active arthropathies (osteoarthritis and acute pseudogout). After three weeks acclimation in culture to eliminate non-fibroblast-like cells, ROS production by the primary human synovial fibroblast-like cells was quantified with live cell imaging of DCF fluorescence after activation with TRPV1 agonist, resiniferatoxin. Fluorescent imaging (Fig. 4E) and the quantitative graph (Fig. 4F) show typical fluorescence signal acquired from (a) one non-responsive and (b) one responsive synoviocyte out of 85 simultaneously recorded cells. Resiniferatoxin (100 nM) was added in bath perfusion for 4 min at the time indicated by arrow on the graph. The resiniferatoxin initiated ROS production in 42% of the synoviocytes tested in the primary cultures harvested from the clinic patients with active arthropathies.

### ROS Donors Generate ROS and Increase TRPV1 in Synoviocytes

**Figure 5:**
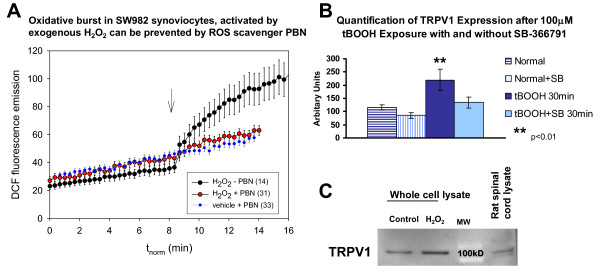
**ROS Activation Increases TRPV1**. **A - H_2_O_2 _Oxidative Burst Is Blocked by PBN**. An oxidative burst in SW982 synoviocytes was activated by the application of H_2_O_2 _(1 mM, arrow) to the media (upper trace). The generated ROS were detected with live cell imaging of the oxidative conversion of the ROS sensitive CM-H2DCFDA fluorophore to DCF over time (min). Presence of the ROS scavenger PBN (2 mM for 2 h, middle trace) in the media prevented the oxidative burst and detected ROS levels were similar to the spontaneous levels seen with the addition of vehicle (with PBN, lower trace). The small error bars are obscured by the large symbols. Numbers of cells are indicated in parenthesis in the legend imaged in three experiments. **B - ROS Induced Increase in TRPV1**. Immunostaining for TRPV1 in SW982 synoviocytes was significantly increased (arbitrary units) compared to control cultures within 30 min after treatment with the ROS donor, tBOOH. The TRPV1 antagonist, SB-366791, inhibits the staining increase. The effect of the tBOOH when combined with the SB-366791 (30 μM) was not significantly different from the controls. All experiments were done in triplicate. *p < 0.01 compared to the control cultures. **C - **The TRPV1 expression increase was measureable by Western blot analysis at 8 h. The protein level (normalized with β-actin) in SW982 synoviocytes was increased almost 2-fold after pulsing with 1 mM H_2_O_2 _for 10 min. All experiments were done in triplicate.

For comparison, ROS production over time after incubation with H_2_O_2 _was tested as shown in Fig. 5. An oxidative burst activated by H_2_O_2 _(1 mM) was prevented when cells were pretreated with the ROS scavenger, PBN (2 mM, 2 h). The average DCF fluorescence (± s.e.m.) was plotted from control (Fig. 5A, lower trace) cells pretreated with PBN as well as from H_2_O_2 _activated cells, with and without PBN (Fig. 5A middle and upper trace, respectively). Treatment with the ROS scavenger alone had no effect on the synovial cell cultures beyond the gradual spontaneous oxidation of the dye seen over time in vehicle treated cells (Fig. 5A, lower trace).

Activation of synoviocytes with the ROS donor, tBOOH, increased the immunocytochemical staining intensity for TRPV1 almost two-fold (1.9×) within 30 min (Fig. 5B) (control 115.6 ± 10.5 versus activated 219.8 ± 41.49 arbitrary units, p < 0.05). This increase was blocked by the TRPV1 antagonist, SB-366791 (30 μM)(134.5 ± 20.55). The increase in TRPV1 protein was measureable with Western blot at 8 h normalized with β-actin controls (Fig. 5C).

### TRPV1 and ROS Generate COX-2, RANTES, TNFα and Additional TRPV1

**Figure 6:**
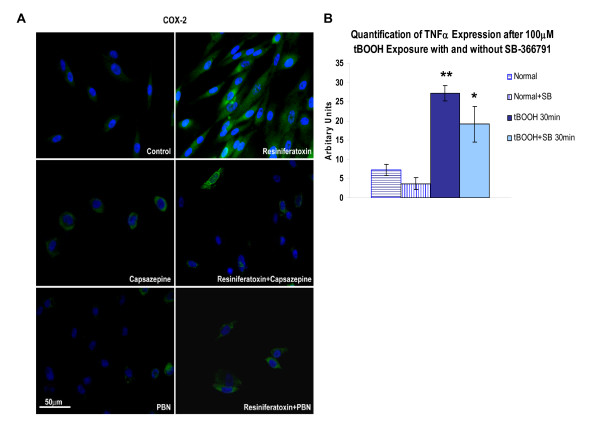
**TRPV1 and ROS Mediated Increase in Inflammatory Mediators**. **A - COX-2 Staining Increase Is Blocked by ROS Scavenger PBN in SW982 Synoviocytes**. Clonal SW982 synoviocytes are activated by TRPV1 agonist, resiniferatoxin, resulting in an increase in immunostaining for inflammatory mediator producing enzyme, COX-2. The COX-2 expression was blocked by either the TRP agonist capsazepine or the ROS scavenger, PBN, while the blockers alone had no effect. All experiments were done in triplicate. **B- ROS Donor Induced TNFα Expression Increase Is Inhibited by TRPV1 Antagonist, SB-366791**. Synoviocytes activated by ROS donor, tBOOH, also have increased immunoreactivity for the inflammatory mediator, TNFα, within 30 min that is significantly increased over control cultures. The effect of the tBOOH was reduced slightly by the TRPV1 antagonist, SB-366791. All experiments were done in triplicate and intensity measurements (arbitrary units) determined with the MetaVue computer-assisted imaging system.* p < 0.05 and ** p < 0.001 compared to the control cultures.

Stimulation of human clonal SW982 synoviocytes with resiniferatoxin, greatly increased immunocytochemical staining for the inflammatory enzyme COX-2 (Fig. 6A) and the chemokine RANTES (not shown) visualized at 24 h. The TRPV1 induced increase in COX-2 staining was reduced by pre-treatment with either TRPV1 antagonist capsazepine (5 μM) or the ROS scavenger PBN (2 mM). Neither inhibitor had an effect on the clonal synoviocytes when applied in otherwise unstimulated cells. Significantly increased staining for TNFα was also evident within 30 min in synoviocytes activated with ROS donor tBOOH (100 μM) (Fig. 6B). The increase in TNFα levels after activation with the ROS donor was less in the presence of the TRPV1 antagonist SB-36791 (30 μM).

## Discussion

### ROS and TRPV1 as Initiators of Neurogenic Inflammation Induced Hypersensitivity

#### Central Sensitization

TRPV channels respond to thermal, chemical, pH, osmotic, and mechanical stimuli, with an inward current carried predominantly by calcium and sodium ions. All of these TRPV1 channel activators are abundant in inflamed and injured tissue, as are ROS. The current findings indicate that the ROS scavenger, PBN, normalizes the inflammation induced behavioral responses tested on the footpad of the affected hindlimb in the knee joint k/c monoarthritis model. Thus, inhibition of ROS generation centrally and perhaps peripherally improves the adverse nociceptive outcomes in this model.

The generation of excess ROS in the spinal cord is observed histologically in close association with the incoming large myelinated afferent nerve fiber bundles. Glial cell generation of ROS would provide a means of direct sensitization of myelinated afferent nerve fibers locally in the dorsal horn, extending the direct influence of the afferent nerves bringing nociceptive input to the spinal cord from the knee joint. Knee joint afferents are reported to be primarily unmyelinated fibers [22,23]. It is likely that ROS contribute to the primary afferent and spinal cord mechanisms detected as secondary thermal and mechanical sensitization by testing the footpad of the animals with knee joint inflammation in this and a previous study [14]. ROS scavengers have been shown to reduce the spinal cord long-term potentiation mechanism that contributes to central sensitization [24].

#### Peripheral Sensitization

In the current study, we have shown that TRPV1 activation of local synoviocytes is also highly relevant to the pathophysiology in inflamed joints through interactions with reactive oxygen species. These processes include the mirrored increase in blood flow likely responsible for bilateral knee joint temperature increase in this arthritis model. Capsaicin mediated increase in peripheral blood flow has been shown to be reduced in TRPV1 knockout mice [15]. Experiments have shown capsazepine inhibits the release of calcitonin gene-related peptide (CGRP) from cardiac sensory nerves that physiologically respond to a decrease in pH of the perfusion solution in isolated heart preparations [25,26]. This CGRP release along with substance P initiates protein/plasma extravasation and edema, increased blood flow and resultant temperature increase.

The overall conclusion of the *in vivo *study is that ROS themselves act in sensitization processes contributing to further inflammation and increased nociception. These findings contribute to an emerging concept that elevated levels of ROS play a critical role in the peripheral and central sensitization underlying persistent pain and are potentially TRPV1 mediated. To further address and support this concept in the context of the local peripheral microenvironment in inflamed joints, further studies with *in vitro *synoviocyte models were done that provided evidence of reciprocal interactions between TRPV1 activation and ROS generation.

### TRPV1 Mediated ROS Generation in Synoviocytes

We have previously identified, characterized and sequenced TRPV1 and TRPV4 isoforms on clonal and primary human synoviocytes [18]. Live cell imaging of calcium mobilization in the synoviocyte cultures was conducted in the presence of TRPV1 agonists and rapid changes in bath temperature or pH. In the current study, live cell imaging of synoviocyte cultures with a ROS sensitive dye indicated that TRPV1 agonists can elicit ROS elevations. ROS generation in clonal synoviocytes was observed in response to TRPV1 agonists capsaicin and resiniferatoxin, and hydrogen peroxide. These enhanced ROS responses were blocked by a TRPV1 antagonist or ROS scavenger, PBN, indicating that ROS generation in synoviocytes is TRPV1 mediated in the experimental conditions utilized.

### TRPV1-Mediated ROS Increases Inflammatory Mediators

Inflammatory products are produced locally in inflamed joints by activated synoviocytes. In the current study, we have detected activation responses mediated by TRPV1 and ROS that include increases in COX-2, RANTES and TNFα. An increase of COX-2 expression in synoviocyte cultures was blocked by either PBN or a TRPV1 antagonist. The ROS induced increase in TNFα was blocked by the ROS scavenger.

Conversely, ROS production in synovial fibroblasts harvested from patients has been shown in a previous study to be increased significantly by IL-6 and inhibited by methotrexate [27]. Modulation of cytokine synthesis and ROS production may contribute to the therapeutic effects of the anti-arthritic agent, methotrexate. These results suggest that the inflammatory events in the synovium of patients with rheumatoid arthritis are augmented locally by ROS generation and the subsequent production of cytokines. ROS released by chondrocytes during inflammation of the synovial membrane have also been associated with cartilage degradation in osteoarthritis (for review see [28]).

We also report here that synoviocytes increase TRPV1 protein expression after administration of ROS donors. We have shown previously that TNFα induces increases in TRPV1 in synoviocytes [19], and that TRPV1 activation increases TNF receptors (TNFR1) in dorsal root ganglia (DRG) through a ROS mediated signalling pathway [29].

It is well known that inflammatory mediators and products (Ex. TNFα, COX-2) can directly sensitize peripheral nerve endings [30-37], suggesting a potential direct interaction between synoviocytes and peripheral nerves. A metabolite of COX-2, prostaglandin E2, is a components of the "inflammatory soup" used in many studies to activate peripheral nerves and was used combined with low pH in studies by Peter Reeh and colleagues [38]. Bolyard and colleagues have found that continuous exposure of rat sensory neurons to "inflammatory soup" results in continued sensitization [39]. We have found previously that afferent innervation contributes to knee joint inflammation since cutting dorsal roots or spinal cord block of glutamate receptors and dorsal root reflexes reduce inflammation in half [20,40-42]. It has been shown previously by others that desensitization of TRPV1 in the knee joint with millimolar doses of resiniferatoxin reduces hyperalgesia, locomotor effects and joint circumference in a similar knee joint inflammation model [43]. These findings suggest that tissue inflammation and injury would activate TRPV1 ion channels and excess ROS would be generated to increase nociceptive responses and other inflammatory response processes.

Other studies have shown that capsaicin acting through TRPV1 generates ROS production in several cell types, including breast epithelial, hepatoma and embryonic kidney cells [1-3], but TRPV1 may suppress ROS formation in glioblastoma cells [4]. Both TRPV1 and P2X ion channels mediate the sensory transduction of ROS, especially H_2_O_2 _and hydroxyl radicals (•OH), by capsaicin-sensitive vagal lung afferent fibers [5]. Evidence from several studies has shown that free oxygen radicals stimulate sensory nerve endings through direct actions on capsaicin activated ion channels on primary afferent nerve endings [6,24,44-46]. These studies have shown that administration of capsazepine or a nonspecific TRPV blocker, ruthenium red, prevents afferent nerve activation by free oxygen radicals. Increased primary afferent nerve activity is known to evoke central sensitization as seen following capsaicin injection [47], peripheral injury [48], or inflammation [49]. Importantly, these previous studies have demonstrated that administration of capsazepine has no effect on the baseline activity of either vagal or sympathetic cardiac afferents, but does abolish the response of afferent nerve fibers to capsaicin, H_2_O_2_, and xanthine + xanthine oxidase.

## Conclusions

The data shown here illustrate that central sensitization and increased temperature responses in an experimental monoarthritis model can be limited by systemic administration of a ROS scavenger. Spinal cord ROS contributing to the sensitized behavioural responses were concomitantly reduced by the scavenger. Examination of human clonal and primary synoviocytes *in vitro *in the current study found, more importantly, that TRPV1 activation represents an important potential peripheral mechanism initiating the generation of ROS and the inflammatory products, COX-2 and TNFα, by the resident synoviocytes that are known to proliferate in arthritic joints. Reciprocally generated increases in ROS, TRPV1 activation and proinflammatory products by synoviocytes were detected after an induced oxidative burst or TRPV1 activation suggesting a potential mechanism for conversion to a more chronic state of inflammation and pain. Together these exciting data implicate TRPV1 ion channels and ROS as interactive partners in neuroinflammatory processes both centrally and peripherally. Their interactions lead to intensification of nociceptive responses and inflammatory responses with potential to damage inflamed joints. Thus, the data support the benefits of agents limiting or eliminating the increases in TRPV1 mediated ROS as a novel adjuvant therapy for more comprehensive treatment of the persistent pathologic pain and inflammation of arthritis.

## Methods

All experimental research was performed under protocol approved by the Institutional Animal Care and Use Committee. All experimental research followed institutional, national and internationally recognized guidelines. All animals were housed in AAALAC and USDA approved facilities.

### Induction of Arthritis and Intrathecal ROS Indicator Dye Injection

All of the rats were anesthetized with isoflurane and polyethylene tubing inserted through the foramen magnum intrathecally to the T10 level of the spinal cord for intrathecal injection of the ROS sensitive dye, DHE (dihydroethidium, Sigma; 50 μl in saline:DMSO, 1:1 injected intrathecally). The following day, half the animals were injected i.p. with the ROS scavenger PBN (100 mg/kg, Sigma) and the other half injected i.p. with saline. One hour later, the animals were anesthetized and their left knee joint injected with 3% carrageenan (plant product and irritant) and 3% kaolin (clay silt) (100 μl in sterile saline) to produce an acute inflammation (4-8 h) confined to the knee joint [20]. Inflammation developed in the knee joint within 4 h, and pain related behaviour was assessed.

### Assessment of Nociceptive Responses and Inflammation

Baseline measurement of circumference, knee joint temperature and nociceptive behaviours were recorded in male Sprague Dawley rats (250-350 g, n = 8), which served as their own controls in a repeated measures intra-subject experimental design. Experimental measures for comparisons of inflamed vs normal joints in rat included:

(1) Assessment of knee joint circumference and temperature of the skin over the lateral surface of the knee. Knee joint temperature was assessed in rats with a small thermoprobe placed on the skin over the knee joint attached to a thermometer with rapid digital readout (Digi-Thermo, Fisher). Random skin sites confirmed that only the knee joint temperature was elevated, bilaterally. Core body temperature was measured with a rectal thermometer. (2) Pain related nocifensive behavioral assessment of nociceptive sensitization was done with hotplate and von Frey fiber tests, standard tests of thermal and mechanical sensitivity. The rats were placed into the Analgesiometer hotplate device with a constant floor temperature of 50°C. Animals were immediately removed when they displayed a flinching, paw flipping or stepping behaviour. A maximal time limit of 20 sec was set when unresponsive animals would be removed from the setup.

For mechanical sensitivity testing, animals were acclimated for 30 min to a cubicle placed on a wire mesh table top. Paw withdrawal responses to two von Frey monofilaments (10 and 47 mN, Semmes-Weinstein Anesthesiometer Kit #18011, Wood Dale, IL), were recorded two days before induction of arthritis and 4 h after the induction by an observer who was blind to the animals' condition. A single trial consists of 10 applications of the filament applied once every second. Trials were repeated no less than 3 min apart to allow the rat to cease any response and return to an inactive posture. Data are presented as a mean response (1-10) and plotted against time after induction of arthritis. An increase in response frequency reflects secondary mechanical allodynia for the 10 mN monofilament and secondary mechanical hyperalgesia for the 45 mN monofilament.

### Detection of ROS in Tissue Sections

ROS in the spinal cord dorsal horn were visualized using a detector dye dihydroethidium (DHE, Molecular Probes, Eugene, OR). The dye is converted to a stable fluorescent product by ROS and allows visualization of ROS accumulated in spinal cord of rats during the time after knee joint inflammation. The DHE (50 μl, 100 mM in DMSO:saline, 1:1) was injected intrathecally through the foramen magnum of anesthetized rats onto the lumbar level of the spinal cord with a blunt 30 G needle attached to a 250 μl Hamilton syringe. Animals were allowed to recover and knee joint inflammation was induced 24 h later. The tissue stable fluorescent ROS indicator DHE was visualized in aldehyde fixed and cut spinal cord at the conclusion of the behavioral study, i.e. 6 h after induction of arthritis. Animals were anesthetized with an injection of Sleepaway pentobarbital solution and transcardially perfused with 0.9% heparinized saline (500 ml) followed by 4% phosphate buffered paraformaldehyde (1000 ml). The spinal cord was dissected, cryoprotected overnight in 30% buffered sucrose and frozen sectioned (15 μm). Spinal cord sections were cut and mounted on gel coated slides and DHE fluorescence visualized with rhodamine filter settings on a Nikon E1000 photomicroscope equipped with the MetaVue Optical Imaging system.

### Human Clonal and Primary Synoviocyte Cultures

#### Clonal Synoviocytes

A clonal fibroblast-like cell line SW982, derived from a human synovial sarcoma, was obtained through American Type Cell Collection (ATCC, Bethesda MD, #HTB-93). Immunocytochemical studies have ascertained that these cells are vimentin positive, smooth muscle actin positive and CD11b negative, consistent with the phenotype of type B synovial cell fibroblasts [19]. In addition the SW982 cells did not stain with anti-neuronal nuclei (neuN) antibody.

Clonal cells were maintained and grown in Leibowitz medium containing 10% heat-inactivated fetal calf serum, 2 mM L-glutamine, 1000 U/ml penicillin G and 1000 pg/ml streptomycin (Invitrogen, Carlsbad, CA or Gibco, Grand Island, NY). Thirty percent conditioned media was retained at each fresh media change. Cells were maintained in a humidified cell incubator at 37°C and 5% CO_2_. Cells were split using 0.25% trypsin/0.02% EDTA for disruption and plated in a 24 well plate on glass slides at a density of 20-40,000 cells per well for immunocytochemistry or plated on 15 mm circular quartz glass coverslips at a density of 200,000 cells/mm^3 ^for live cell imaging. Clonal cells were used in passages 4-21.

#### Primary Synoviocytes

Primary cultures of surface-adherent synoviocytes were established from synovial fluid derived from the discarded sample of a clinical arthrocentesis procedure performed on patients with acute pseudogout or osteoarthritis, based on American College of Rheumatology criteria and in accordance with the guidelines and approved protocol of the University of Texas Medical Branch Institutional Review Board. Cells were maintained in DMEM, 10% heated FBS, 2 mM L-glutamine, and penicillin-streptomycin. Primary cultures were incubated in a humidified cell incubator (37°C with 5% CO_2 _atmosphere) for 3 wk before testing. Cells were split with 0.25% trypsin-0.02% EDTA for disruption and plated on 15 mm circular quartz glass coverslips at a density of 200,000 cells/mm^3^.

### Real-Time Live Cell Imaging of ROS

In the 24-48 h prior to experiments, cells were harvested and plated onto 15 mm quartz glass coverslips. Standard bath solutions consisted of (in mM) 150 NaCl, 5.5 KCl, 1 MgCl_2_, 4 CaCl_2_, 5 glucose, and 10 HEPES, adjusted to pH 7.4 at RT with NaOH (330 mosmol/l). On the day of the experiment, primary or SW982 synoviocytes were pre-loaded with a ROS-sensitive dye, 2,7-dichlorofluorescein diacetate (DCFDA, 2-4 μM for 30 min at a room temperature, Molecular Probes, Invitrogen, Eugene, OR), a non-fluorescent chemical oxidized intracellularly by ROS to the highly fluorescent derivative DCF. After pre-loading, the cells were washed in a physiological saline solution, allowed to stay from 30 min to 2 h at a room temperature, placed in a continuously superfused thermoregulated chamber (Warner Instruments, Hamden, CT) and then mounted to the stage of inverted Nikon Diphot microscope for recording. Application of chemicals onto individual cell was achieved by gravity flow from a large-bore pipette placed 300-400 μm from the cell. The dye-loaded cells were excited at 488 nm and emitted light collected using 490 nm long pass, or 535 nm (bandwidth 40 nm) filter. Real-time live cell imaging at high power was done with a Polychrome II monochromator (TILL Photonics, Munich, Germany) controlled by X-chart software (HEKA, Heidelberg, Germany) and with an ITC-18 computer interface (Instrutech, Port Washington, NY) for single cell fluorescence excitation. The resulting DCF emissions were detected with a Hamamatsu R928 photomultiplier. Imaging was performed in Dubecco's PBS (pH 7.4, RT) using a Nikon TE2000-S Eclipse quantitative fluorescence live-cell imaging system equipped with a high sensitivity 12 digital monochrome cooled Coolsnap ES CCD camera (Photometrics, Tucson, AZ). Lambda LS illumination and a Lambda 10-B Smart Shutter (Sutter Instruments, Novato, CA) were controlled by Metafluor software (Molecular Devices, Sunnyvale, CA). Metamorph (Molecular Devices, Sunnyvale, CA) software was used for digital image processing, and Excel scientific software (SPSS, Chicago, IL) was used for conversion and analysis of acquired data. Experiments were repeated a minimum of three times each.

Preincubation of the SW982 cells with TRPV1 antagonists capsazepine (5 μM, Calbiochem, La Jolla, CA), SB-366791 (30 μM, Tocris, Ellisville, MO) or ROS scavenger, PBN (2 mM, Sigma, Chemical, St. Louis, MO) was performed 2 h before addition of resiniferatoxin (100 nM) or capsaicin (1 μM, Tocris, Ellisville, MO). After application of resiniferatoxin, the cells were incubated for 2 h at 37°C in an incubator at ambient CO_2_. After 2 h, the cell culture supernatant was aspirated and replaced with regular media or with regular media containing capsazepine or PBN at concentrations noted above. The cultures were then incubated overnight (37°C, 5% CO_2_) prior to removal of supernatant for Western blot analysis of ROS metabolites and immunocytochemical localization of TRPV1, COX-2, and TNFα.

### Measurement of ROS metabolites by HPLC

As an indicator of TRPV1 mediated ROS released into the supernatant, ROS metabolites 2,5-DHBA and 2,3-DHBA were trapped with salicylic acid [50] and the samples analyzed by high performance liquid chromatography with electrochemical detection (HPLC-ECD). Given that the hydroxyl radical is a short-lived, highly reactive chemical, it cannot itself be collected and preserved in a sample tube. The salicylic acid trapping agent (2.5 mM) reacts with the hydroxyl radical to produce DHBAs. The level of hydroxyl radical production in response to the TRPV1 activation was compared to that in media collected from control cultures from three experiments. Comparison of the results based on the quantities of increased 2,3-DHBA were considered reliable because there is some endogenous enzymatic formation of 2,5-DHBA.

### Immunocytochemical Detection of TRPV1, COX-2 and TNFα in SW982 Synoviocytes

After treatments, the cells on glass coverslips were washed with phosphate buffered saline (PBS, with 1% bovine serum albumin (BSA) and 0.9 mM CaCl_2 _and 0.5 mM MgCl_2_) and fixed with a mixture of methanol and 4% freshly mixed paraformaldehyde (1:1, v/v) for 10 min at room temperature. The coverslips were washed and incubated at room temperature for 30 min with 3% nonspecific species serum. The primary antibody (1:1,000 anti-TRPV1, Alomone, Isreael; 1:1,000 anti-COX-2, BD Transduction Labs San Diego CA; 1:500 anti-TNFα, R&D Systems, Minneapolis, MN) was diluted in PBS with 1.0% normal goat serum (NGS, Sigma Chemical). The primary antibody was allowed to incubate on the glass coverslips for 48 h at 4°C, or overnight at room temperature. The glass coverslips were then washed and incubated for 30-60 min at room temperature with the appropriate species secondary IgG antibody with a fluorescent ALEXA tag (1:1000 dilution, Alexa Fluor 588 green; Molecular Probes, Inc. Eugene OR). After final washes, the stained coverslips were inverted onto a dot of mounting media containing nuclear counterstain DAPI (VectaShield Hardset, Vector Labs, Burlingame, CA). Immunocytochemical controls included the absence of the primary or secondary antibodies, or were done in the presence of matched serum at the same dilution. The specificity of the antibodies was confirmed for TRPV1 by adsorption controls with P-19 N-terminus peptide provided by Santa Cruz, and for TRPV4 with peptide 853-871 supplied by Alomone Labs (1:1 w/w, 30 min). There was no staining in either of the method controls or the peptide blocked controls. ROS donor tBOOH (100 mM) stimulated increase in TRPV1 was greatly reduced by co-incubation with a TRPV1 inhibitor, SB-366791 (30 mM). Stained cells were visualized with a Nikon FXA microscope equipped with a Coolsnap digital low light camera and MetaVue image capture software (Nikon Instruments, Inc. Melville, NY).

### Western blot analysis

Low passage SW982 cell cultures were grown in 75 mm flasks until they reached 70-80% confluency. Proteins were extracted from control cultures without treatment and after treatment with H_2_O_2 _(1 mM) for 8 h. Western Blot analysis was performed on whole cell protein fractions extracted from synovial SW982 cells using the ProteoExtract Subcellular Proteome Extraction kit (Calbiochem, Cincinnati, OH). Proteins transferred to membranes were probed with anti-TRPV1 antibody (VR1, P-19, 1:1000, Santa Cruz, Burlingame, CA) at 4°C overnight. Amounts of total protein loaded per lane included: 15 μg for cytosolic and membrane fractions, 10-15 μg for whole cell and 5 μg for rat spinal cord lysate as a positive control. Western blot analysis was performed with samples from three experiments.

### Power Analysis and Statistical Tests

Statistical analyses for behavioural studies were performed using Prism software. Descriptive statistics for these data are expressed as mean ± S.D. Behavioral data were compared with non-parametric repeated measures analysis of variance using Mann-Whitney U post-hoc tests unless otherwise specified. For measurements of ROS concentrations, the overall percent change from baseline values over time and differences between groups were compared with a multivariate analysis of variance (MANOVA). Sigma Plot and Sigma Stat scientific software (SPSS, Chicago, IL) were used for conversion and analysis of acquired data. Data are reported as means ± SEM. Within subjects, comparisons were made for baseline, time of injection peak, and time of peak increase. All statistical comparisons were evaluated at an alpha level of significance of 0.05.

## Abbreviations

**COX-2**: cyclo-oxygenase 2; **DCFDA**: 2,7-dichlorofluorescein diacetate; **DHE**: dihydroethidium; **DHBA**: dihydroxybenzoic acid; **DPBA**: dipeptide boronic acid; **k/c**: kaolin/carrageenan; **PBN**: *N*-*tert*-Butyl-α-phenylnitrone; **ROS**: reactive oxygen species; **SF**: synovial fluid; **TRP**: transient receptor potential;

## Competing interests

The authors declare that they have no competing interests.

## Authors' contributions

KNW designed the animal study, wrote the manuscript and produced the graphics; MYK and KNW designed and performed the ROS live cell imaging studies; TAM designed and performed the *in vitro *synoviocyte staining studies; YL performed the animal behavioral studies, histology and photography. All authors read and approved the final manuscript.

## Authors' information

Dr. Terry McNearney is currently employed at Eli Lilly and Co., Indianapolis IN 46285. Dr. Mikhail Kochukov is currently employed at Baylor College of Medicine, Houston TX 77030.
